# Detection of wheat *Fusarium* head blight using UAV-based spectral and image feature fusion

**DOI:** 10.3389/fpls.2022.1004427

**Published:** 2022-09-21

**Authors:** Hansu Zhang, Linsheng Huang, Wenjiang Huang, Yingying Dong, Shizhuang Weng, Jinling Zhao, Huiqin Ma, Linyi Liu

**Affiliations:** ^1^National Engineering Research Center for Agro-Ecological Big Data Analysis and Application, Anhui University, Hefei, China; ^2^State Key Laboratory of Remote Sensing Science, Aerospace Information Research Institute, Chinese Academy of Sciences, Beijing, China; ^3^University of Chinese Academy of Sciences, Beijing, China; ^4^Key Laboratory for Earth Observation of Hainan Province, Sanya, China

**Keywords:** hyperspectral images, UAV, crop stress, feature fusion, classification models

## Abstract

Infection caused by *Fusarium* head blight (FHB) has severely damaged the quality and yield of wheat in China and threatened the health of humans and livestock. Inaccurate disease detection increases the use cost of pesticide and pollutes farmland, highlighting the need for FHB detection in wheat fields. The combination of spectral and spatial information provided by image analysis facilitates the detection of infection-related damage in crops. In this study, an effective detection method for wheat FHB based on unmanned aerial vehicle (UAV) hyperspectral images was explored by fusing spectral features and image features. Spectral features mainly refer to band features, and image features mainly include texture and color features. Our aim was to explain all aspects of wheat infection through multi-class feature fusion and to find the best FHB detection method for field wheat combining current advanced algorithms. We first evaluated the quality of the two acquired UAV images and eliminated the excessively noisy bands in the images. Then, the spectral features, texture features, and color features in the images were extracted. The random forest (RF) algorithm was used to optimize features, and the importance value of the features determined whether the features were retained. Feature combinations included spectral features, spectral and texture features fusion, and the fusion of spectral, texture, and color features to combine support vector machine, RF, and back propagation neural network in constructing wheat FHB detection models. The results showed that the model based on the fusion of spectral, texture, and color features using the RF algorithm achieved the best performance, with a prediction accuracy of 85%. The method proposed in this study may provide an effective way of FHB detection in field wheat.

## Introduction

Wheat is the second largest grain crop in China. The stable and high yield of wheat has been the focus of agricultural production (Huang et al., 2020). *Fusarium* head blight (FHB), also known as scab, is a devastating wheat disease caused by the fungal plant pathogen *Fusarium graminearum* (*Gibberella*). *Fusarium*-infected wheat typically results in small, low mass, and shrunken grains, which can rapidly lead to very large crop losses and quality degradation (Bauriegel et al., 2011b). Furthermore, the fungus produces a large number of mycotoxins (deoxynivalenol, nivalenol and zearalenones etc.), among which the most toxic deoxynivalenol (DON) can disrupt normal cell function by inhibiting protein synthesis, posing a significant threat to human and animal health (Barbedo et al., 2015). In recent years, with global climate change, wheat FHB infection has become increasingly serious, resulting in severe damage to wheat quality and yields. Ineffective FHB management practices hinder the profitable and sustainable production of wheat, affecting its economic and social benefits in China. Therefore, the detection of disease development of wheat is important and essential for successful disease control.

Traditional FHB detection mainly relies on professionals to scout the development of wheat infection through visual interpretation, or scholars use chemical methods, such as gas chromatography (GC) (Simsek et al., 2012), high performance liquid chromatography (HPLC) (Simsek et al., 2012), enzyme-linked immunosorbent assay (ELISA) (Maragos et al., 2006), and polymerase chain reaction (PCR) (Amar et al., 2012; Atoui et al., 2012) to detect FHB and DON production. However, these methods are time-consuming, labor-intensive, unable to achieve large-scale monitoring, and are destructive to wheat. Remote sensing technology has been widely used in the monitoring and identification of wheat FHB with nondestructive inspections and rapid measurements. At present, monitoring of wheat FHB using remote sensing technology is mainly manifested in three aspects: (i) identify wheat kernels with varying degrees of damage under laboratory conditions to accurately judge the quality of wheat kernels (Delwiche et al., 2011; Barbedo et al., 2015; Jaillais et al., 2015; Alisaac et al., 2019; Femenias et al., 2020; Liang et al., 2020; Zhang D. Y. et al., 2020a; Zhang D. Y. et al., 2020b); (ii) use remote sensing technology to capture the information of individual or canopy wheat infected with FHB to accurately detect the disease (Dammer et al., 2011; Menesatti et al., 2013; Whetton et al., 2018a,b; Huang et al., 2019b; Zhang et al., 2019; Huang et al., 2020; Ma et al., 2020; Huang et al., 2021); and (iii) monitor wheat FHB on a regional scale with remote sensing (Liu et al., 2020b). However, there are many limitations in these studies. The inspection of wheat kernels has a time lag that only allows the use of kernels with different qualities and cannot fundamentally ameliorate wheat infection. Quantitative detection studies at the single plant scale or canopy scale only provides a theoretical reference without the spatial distribution of wheat infection to meet the needs of practical applications. Optical satellite images are at risk of being covered by clouds, and FHB may occur severely and frequently in cloudy and foggy areas, reducing the availability of remote sensing images (Liu et al., 2020a). Therefore, there is an urgent need for new technological means to solve the current problems.

Unmanned aerial vehicles (UAVs) are considered a practical detection method for crop pests and diseases. Unlike near-ground and satellite-based remote sensing platforms, applications of UAV have the advantages of large coverage, high efficiency, and flexibility (Fu et al., 2022; Zhu et al., 2022a). UAV can collect very high-resolution images and data in a cost-effective manner over a short period of time (Ye et al., 2020). As a new technological means, UAV technology has made significant progress in crop classification, growth monitoring, and identification of pests and diseases. UAV also allows for a proper balance between image quality, sensing efficiency, and operating cost (Li et al., 2019). At present, UAV images are mainly divided into multispectral images and hyperspectral images. Hyperspectral images have dozens to hundreds of continuous and subdivided spectral bands in the ultraviolet, visible, near-infrared, and mid-infrared regions, making them more sensitive to the reflected energy of light and increasingly available (Liu et al., 2020a). Hyperspectral images can provide image and spectral data of each pixel, thus detecting the internal chemical compositions and external phenotypic traits of objects (Zhang D. Y. et al., 2020b). Currently, there are few reports on the detection of FHB infection in wheat using UAV hyperspectral technology (Liu et al., 2020a; Ma et al., 2021; Xiao et al., 2021). We attempted to use UAV hyperspectral technology to explore wheat FHB detection methods in our study. What’s more, scholars have primarily mined spectral features that could characterize physiological and biochemical changes (such as moisture, pigment, etc.), as well as considered texture features that can represent spatial changes of wheat to detect FHB. In fact, the infected wheat tissue usually transitions from green (healthy tissue) to yellow–white (diseased tissue) as the disease progresses. Color has been proven to be the most effective means to distinguish different image objects and realize object recognition among phenotypic traits (e.g., color, texture, and size) extracted from images (Zhang et al., 2018). However, the application of UAV color features in FHB detection has not been explored. Therefore, this study combines color features to further explore the wheat FHB detection methods based on UAV hyperspectral images.

**Figure 1:**
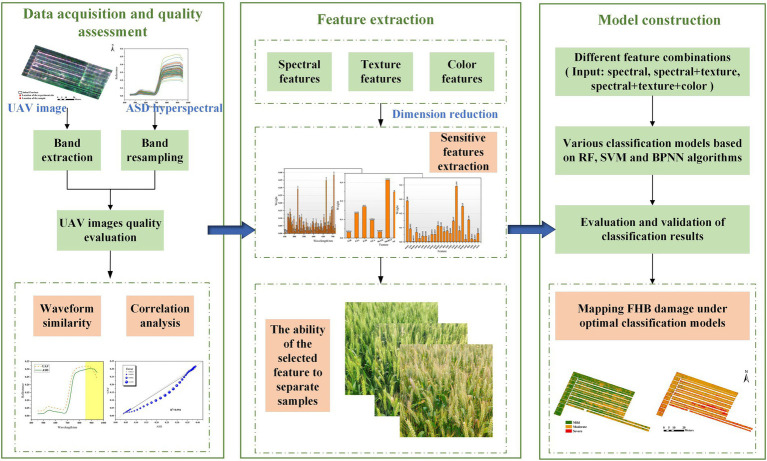
Methodological framework.

Our study investigated the potential of fusing spectral and image features of UAV hyperspectral images to improve the ability of detecting wheat FHB in the field. The overall technical flow chart is shown in Figure 1. First, we determined the most suitable sensitive spectral features to identify FHB; these features reflect the disease stress of the host. Second, we extracted texture features that could represent the disease distribution based on band images containing the most disease information. Finally, we calculated the color features that characterize disease incidence. We combined multiple algorithms to construct classification models and examine the effect of multi-features on the detection accuracy of FHB. Our goals were to (1) evaluate the performance of UAV hyperspectral images in identifying wheat FHB occurrence; (2) evaluate the potential of multi-features in FHB detection; (3) explore the best classification method for UAV images; and (4) map the occurrence of FHB in a wheat field using the optimal model. In general, we developed a novel method for FHB detection based on UAV images, which forms a basis for the precise prevention and control of FHB.

## Materials and methods

### Experiment site and data acquisition

**Figure 2:**
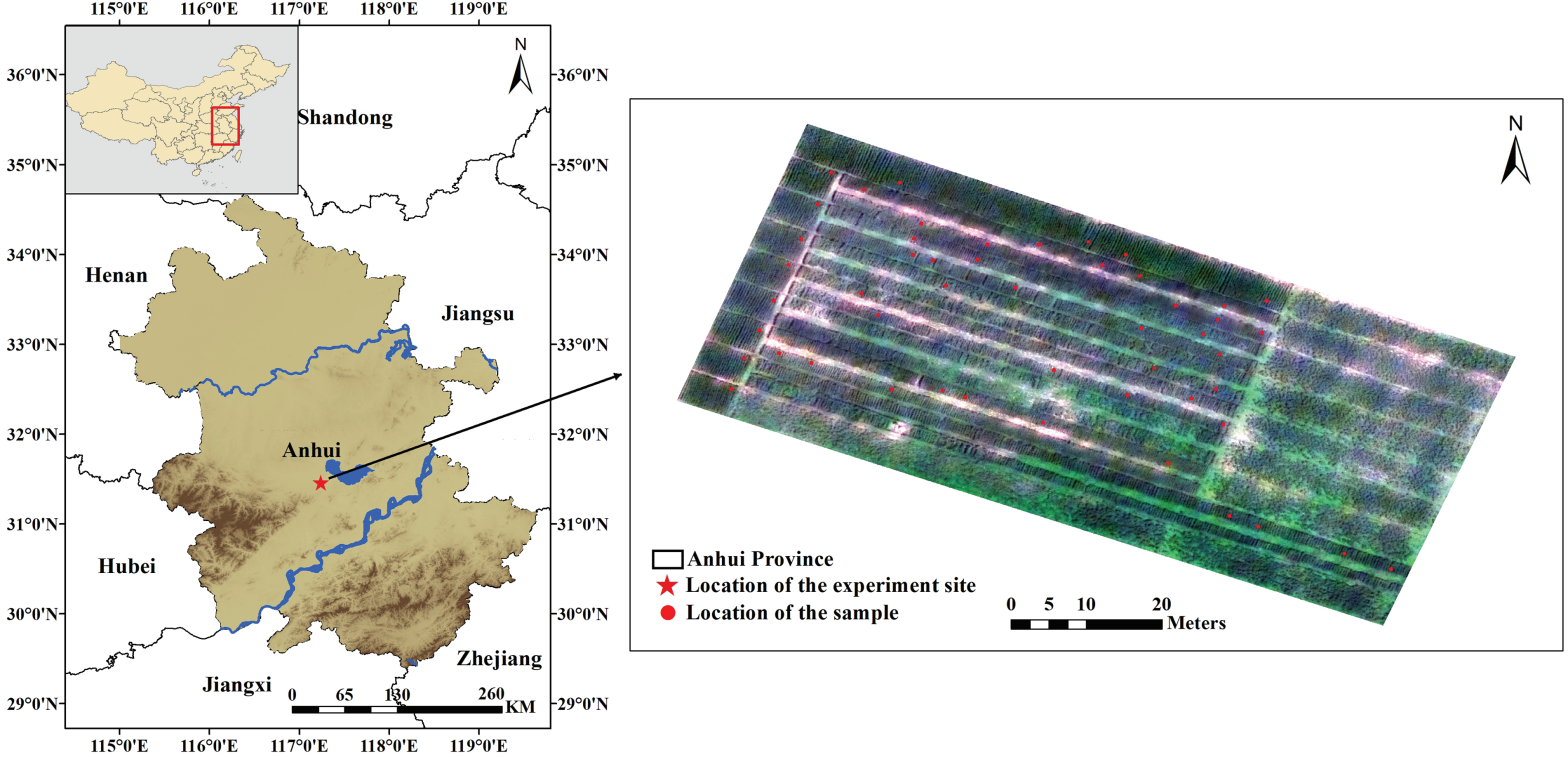
Location of the experiment site and field investigation samples. The star in the left map represents the location of the experiment site, and the right map is the experiment field photographed by the UAV, where red marks the location of the field investigation point.

Our experiment site was situated in the Anhui Agricultural University Production Base (31°290 N, 117°130E) in Lujiang County, Anhui Province, China (Figure 2). The main wheat variety in this area is Yangmai 25, which is susceptible to FHB. Zero tillage and a typical subtropical humid monsoon climate provide favorable conditions for the occurrence of wheat FHB in this region. According to the Anhui Meteorological Service, the average temperature from April to early May 2019 in Lujiang County was about 20°C, accompanied by several days of rainfall. The wheat was in the flowering period in April. Sufficient fungus sources and climatic conditions caused natural wheat FHB in the experiment site.

Data were sourced from UAV image acquisition and field investigation. The UAV images were obtained using an M600 Pro aircraft of Dajang Innovations (DJI) during the wheat filling stage on May 3 and 8, 2019. This system was equipped with a Cubert S185 FireflEYE SE hyperspectral imaging camera (Cubert GmbH, Ulm, Baden-Württemberg, Germany), which can collect the reflected radiation in the 450–950 nm range. The spectral sampling interval was 4 nm, and there were 125 bands in total. The UAV flew at a speed of 3 m/s at an altitude of 60 m. The camera triggers at a frequency of 0.8 s, with a forward overlap of 80% and a side overlap of 65%. All UAV images were collected under clear weather and cloudless skies between 11 a.m. and 1 p.m. (local time). Before capturing hyperspectral images, radiometric correction of the camera was required. A panchromatic image with high spatial resolution and hyperspectral cube image with a low spatial resolution were fused and spliced for subsequent analysis. The final hyperspectral images had a spatial resolution of 4 cm. Field investigation experiments were carried out while capturing the UAV images. Fifty plots (each with an area of 1 m^2^) were evenly selected across the experiment field. These plots were used as ground sample points to verify the quality of the UAV images. To accurately locate the sampling points, we fixed a flagpole next to each point. The canopy spectral reflectance of the sample points was collected using an ASD FieldSpec Pro spectrometer (Analytical Spectral Devices, Inc., Boulder, CO, USA), which has a spectral resolution of 3 nm in the range of 350–1,000 nm and 10 nm in the range of 1,000–2,500 nm. All canopy spectral measurements were carried out at a height of about 1.3 m above the ground, and 10 measurements were taken at each sample point. A BaSO4 calibration panel was used before each measurement to correct for changes in illumination conditions, and the average was used as the final canopy spectrum. According to the rules for monitoring and forecasting wheat head blight suggested by the National Plant Protection Department of China (Chinese Standard: GB/T 15796–2011), the diseased ear ratio (DER) in each plot can be expressed by the ratio of diseased ears to the total investigated ears. The wheat planting density in the study area was relatively uniform. Then, we randomly selected 50 wheat plants at every sample point and recorded the number of diseased wheat plants by visual interpretation. DER was divided into five classes: 0.1% < DER ≤ 10% (Class 1), 10% < DER ≤ 20% (Class 2), 20% < DER ≤ 30% (Class 3), 30% < DER ≤ 40% (Class 4), and DER > 40% (Class 5). Actually, wheat fields with more than 30% infected wheat are severely damaged, and those with less than 10% are mildly damaged. Therefore, we reclassified DER into three grades: mild infection (0.1% < DER ≤ 10%), moderate infection (10% < DER ≤ 30%), and severe infection (DER > 30%) for subsequent analysis.

### Data processing and analysis

#### Data quality assessment of UAV

UAV hyperspectral images are obtained by fusing and splicing a panchromatic image and hyperspectral cube image. UAV is prone to the impact of objective factors such as shaking in flight. Therefore, it is necessary to evaluate the image quality before identifying wheat FHB in the field. ASD spectrometers are widely used in agricultural remote sensing monitoring, and their spectral information is often used as an important basis for monitoring crop pests and diseases (Cao et al., 2013; Ashourloo et al., 2014; Zheng et al., 2018; Huang et al., 2019a; Ma et al., 2020). In this study, we used ASD spectral data as a criterion to evaluate the quality of UAV images (Bareth et al., 2015; Gao et al., 2016; Chen et al., 2018). First, we extracted and averaged the spectral reflectance of all pixels in the sample points to obtain the UAV spectral information in the same region as the ASD measurement. Second, we analyzed the spectral variations between the two data sets by resampling the ASD canopy spectrum and determining the differences in the waveforms. Finally, we calculated the correlation between the resampled ASD spectrum and the UAV spectrum in the 450–950 nm range. If there is a strong correlation between the data measured by the two sensors and the same spectral curve, then the UAV data are considered reliable.

#### Optimal feature selection for wheat FHB detection

The UAV hyperspectral images captured in this study contain 125 spectral bands, from visible to near infrared, which reflect the internal physiological and biochemical changes of wheat after pathogen infection (Li et al., 2014). In addition, wheat presents different spatial distributions as FHB severity increases, as indicated by the texture and color features of an image. Here, we detected FHB of the wheat field by extracting key features from images captured on May 3 and 8. The wheat was at the same growth stage on both dates; therefore, any feature changes between the two dates were mainly due to disease development rather than the wheat growth. It should be noted that the extracted features are not only spectral features but also include image features (texture and color features). Moreover, the extracted features may contain invalid information and thus be insensitive to wheat FHB. A random forest (RF) algorithm was adopted to further reduce data redundancy and develop efficient models.

The spectral features for each plot were extracted from hyperspectral images using the region of interest (ROI) tool in ENVI 5.3 software. The feature extraction method is the same as that used in data quality assessment of UAV. We extracted and averaged the spectral reflectance of all pixels contained in the sample point as the final spectral value of each sample point. The texture features were extracted by the gray level co-occurrence matrix (GLCM) method (Zhang et al., 2017). The GLCM method is a classical statistical analysis technique that describes texture by studying the spatial correlation characteristics of the gray level (Guo et al., 2020). The mean, variance, homogeneity, contrast, dissimilarity, entropy, second moment, and correlation were extracted for FHB detection analysis. Table 1 describes the texture features. Before texture feature extraction, the principal component analysis (PCA) method was used to reduce the dimensionality of the hyperspectral images and generate principal component images containing only three bands. The first three bands contain most of the information (the cumulative variance exceeds 97%); thus, the texture features were extracted from the gray images corresponding to the three bands. The extraction of texture features was completed with ENVI 5.3 software, and the specific process occurred in four steps (Fu et al., 2022): (1) select the gray images in “Texture Input File” dialog, (2) select the necessary texture features in the check box, (3) set the processing window size to 3 × 3 (the smallest window size guarantees the highest resolution), and (4) set the output path and calculate the texture values. A total of 24 texture features were calculated.

**Table 1:** The texture feature used in the study and descriptions.

Texture feature	Abbreviation	Content
Mean	mea	Average of grey levels
Variance	var	Change in greyscale
Homogeneity	hom	Local homogeneity, as opposed to contrast
Contrast	con	Clarity of texture
Dissimilarity	dis	Similarity of the pixels
Entropy	ent	Diversity of the pixels
Second Moment	sem	Uniformity in greyscale
Correlation	cor	Ductility of grey value

**Figure 3:**
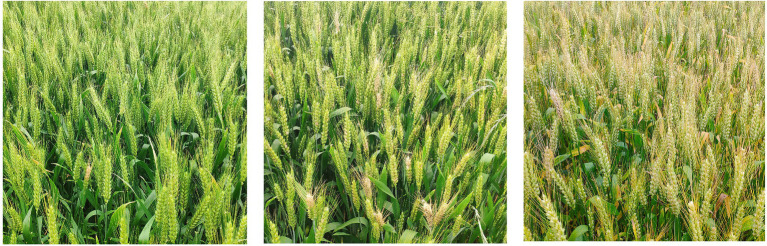
Different incidences of wheat in the field: mild infection (left), moderate infection (center), and severe infection (right).

For the color features selection, we calculated color indices through band combinations to indicate different aspects of wheat infection (Li et al., 2019; Huang et al., 2020; Ge et al., 2021). Color feature is the most widely used visual feature in image retrieval; it is usually related to the object or scene contained in the image; at the same time, color feature is less dependent on the size, orientation, and perspective of the image itself, making it highly robust (Huang et al., 2020). During the mild infection stage, several wheat plants were withered and yellowed in the field. As the infection worsened, the damaged area gradually increased (Figure 3). Chromatic aberration can be used to distinguish the severity of FHB. In this study, three wavelengths (694, 542, and 482 nm) of the hyperspectral images were used to synthesize RGB images and extract color features. The extracted color features mainly included Excess Blue Vegetation Index (ExB), Excess Green Vegetation Index (ExG), Excess Red Vegetation Index (ExR), Green Leaf Algorithm (GLA), Kawashima Index (IKAW), Modified Green Red Vegetation Index (MGRVI), Normalized Green-Red Difference Index (NGRDI), Red Green Blue Vegetation Index (RGBVI), Visible Atmospherically Resistant Index (VARI), and Woebbecke Index (WI). Details of the 10 color features mentioned in this paper are shown in Table 2.

**Table 2:** The color feature used in the study and descriptions.

Color feature (abbreviation)	Full name	Formula	Reference
ExB	Excess Blue Vegetation Index	1.4B-G	Li et al. (2019)
ExG	Excess Green Vegetation Index	2G-R-B	Woebbecke et al. (1995)
ExR	Excess Red Vegetation Index	1.4R-G	Meyer and Neto (2008)
GLA	Green Leaf Algorithm	(2G-R-B)/(2G + R + B)	Louhaichi et al. (2001)
IKAW	Kawashima Index	(R-B)/(R + B)	Kawashima and Nakatani (1998)
MGRVI	Modified Green Red Vegetation Index	(G^2^-R^2^)/(G^2^ + R^2^)	Tucker (1979)
NGRDI	Normalized Green-Red Difference Index	(G-R)/(G + R)	Tucker (1979)
RGBVI	Red Green Blue Vegetation Index	(G^2^-B × R)/(G^2^ + B × R)	Bendig et al. (2015)
VARI	Visible Atmospherically Resistant Index	(G-R)/(G + R-B)	Gitelson et al. (2002)
WI	Woebbecke Index	(G-B)/(R-G)	Woebbecke et al. (1995)

Rational selection of the important features in wheat FHB detection is the most critical step in image analysis. RF consists of multiple decision trees, which can calculate the importance of individual feature variables. The feature evaluation method is called “embedding,” which integrates the features of the filter and wrapper methods (Pal and Foody, 2010). We evaluated the importance of features by calculating the contribution rate of each feature in the random forest, as measured by the Gini index (Deng and Runger, 2013). To reduce the random error generated during the operation of the random forest algorithm, an average of 20 algorithms was set as the final importance score of each feature. Analysis of variance (ANOVA) was used to further test the ability of selected features to separate mild, moderate, and severe disease samples.

### Classification model construction and evaluation

Using MATLAB R2016b (MathWorks, Natick, MA, USA), three algorithms, support vector machine (SVM), RF, and a back propagation neural network (BPNN) were the basis for the detection models of wheat FHB.

SVM is a supervised learning algorithm that realizes the best generalization ability and prevents overfitting by trying to find a compromise between the minimum calibration set error and the maximum edge error; it is one of the most powerful classifiers (Faris et al., 2017). SVM is expected to find an optimal hyperplane to divide the samples and ultimately create a convex quadratic programming problem that only provides global minima (avoiding local minima). When the variables cannot be separated linearly, SVM can use the kernel function to project variables into higher-dimensional feature space, which makes linear division easier (Xia et al., 2016). Compared with other classifiers that require a large number of samples, SVM can find the optimal solution on the basis of existing samples, so it has better applicability to limited samples, lower computational complexity, and less training time. The kernel function, kernel parameter size, and penalty parameter are important factors affecting the performance of the SVM model. We chose the radial basis function as the kernel function and used the grid optimization method to search for the best parameters to obtain better model accuracy.

The RF algorithm, proposed by Breiman (2001), is a popular ensemble learning algorithm in classification, prediction, and feature selection (Breiman, 2001). When using the RF algorithm for classification, the final label of the input sample is determined by voting for each decision tree in the random forest (Guo et al., 2011; Zhu et al., 2022b). Random resampling and node random splitting techniques are used to train the RF model (Gislason et al., 2006). RF is advantageous in remote sensing image processing (Rodriguez-Galiano et al., 2012): (1) RF is less computationally intensive than other tree ensemble methods (such as Boosting) and less prone to overfitting; (2) RF has a strong ability to resist noise and outliers, can tolerate a certain amount of data loss, and has good robustness to noise and outliers; (3) RF can analyze complex classification features and measure the importance of variables; (4) RF supports high dimensional data and generates an internal unbiased estimate of generalization error (“out of bag” error). In this study, the number of model decision trees was set to 200, and other parameters were kept as the default.

BPNN is one of the most widely used network models in remote sensing (Yang et al., 2011). It is a multi-layer feedforward neural network based on error backpropagation algorithm training, usually including an input layer, hidden layer, and output layer. When a set of information is inputted, the network can achieve the target accuracy through continuous repeated training and adjustment so as to produce satisfactory results. The algorithm continuously collects the errors generated by the model during the training period, returns these errors as output values through back propagation, and then continuously adjusts the weight of each neuron according to the error value. Finally, the best classification by the model is achieved.

A total of 100 samples with mild, moderate, and severe disease progression were randomly divided into the calibration set and prediction set (4:1 ratio). The calibration set was used for model construction, and the prediction set was preliminarily used to evaluate the capabilities of the model classification. To further evaluate the accuracy and prevent the model from overfitting, the validation set was used to verify the generalization ability of the model. We employed a five-fold cross-validation method to equally divide the dataset into five parts, each of which was an independent validation set. The accuracy of each validation set was evaluated, and the average was used as the final model validation accuracy. The calibration accuracy, prediction accuracy, and validation accuracy demonstrated the model’s ability to detect wheat FHB in our study. Using ArcGIS 10.6 software to map the damage of wheat FHB, and calculate the ratio of the number of infected pixels to the number of healthy pixels to statistics the proportion of wheat areas with different infection grades.

## Results

### UAV data quality verification based on canopy data

**Figure 4:**
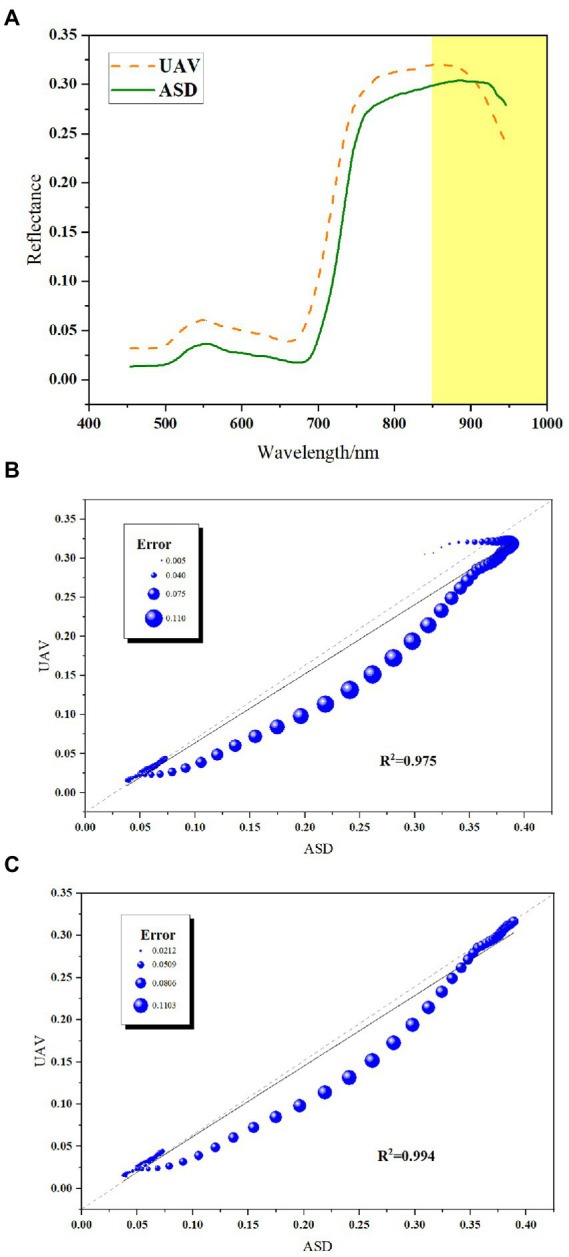
Curve comparison and correlation of UAV and ASD spectra. **(A)** Curves of ASD and UAV spectra. **(B)** Correlation between the two types of curves at 450–950 nm. **(C)** Correlation between the two types of curves at 450–850 nm.

Figure 4A shows the original mean spectrum of canopy wheat measured by the ASD spectrometer and extracted from the UAV images over 450–950 nm. From the perspective of waveform similarity, the variations of the two spectra in the visible to near-infrared region (450–850 nm) are consistent, with significant peaks (near 550 nm) and troughs (near 680 nm). However, the spectrum measured by the ASD spectrometer is lower than the spectrum extracted by the UAV images overall. Above 850 nm, the spectral reflectance of the UAV images gradually decreases, and the spectral curve shows a significant downward trend compared with that of ASD, while the ASD spectral curve has little fluctuation. Figure 4B shows the correlation between UAV spectrum and ASD spectrum in the range of 450–950 nm. The two spectra are highly correlated, with *R*^2^ above 0.97, which indicates that the image quality of UAV is trustworthy. The correlation between the UAV spectrum and ASD spectrum within 450–850 nm was further analyzed: *R*^2^ reached 0.99 (Figure 4C). Thus, the band greater than 850 nm greatly influences the UAV images. Therefore, the last 100 bands of the UAV images were excluded from post-processing.

### Optimal spectral and image features

**Figure 5:**
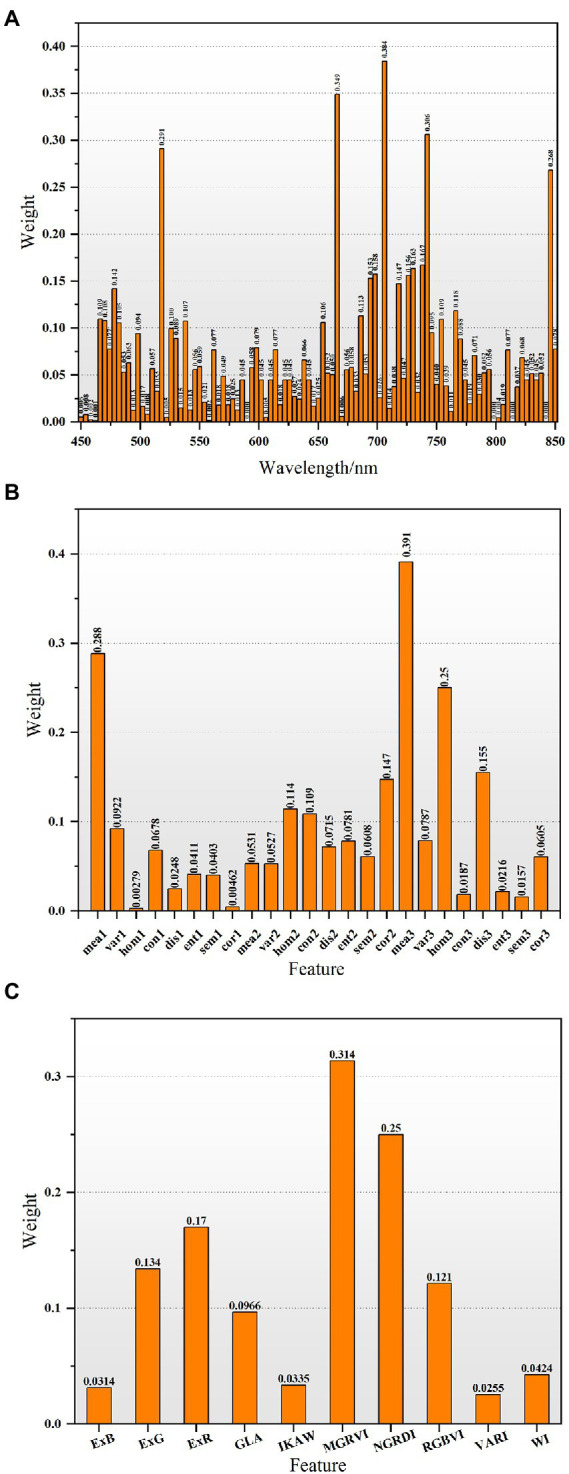
The importance distributions of various features based on the RF algorithm. **(A-C)** represent the weights of spectral features, texture features, and color features, respectively.

The RF algorithm was used to evaluate the importance of each feature in the FHB detection models to filter out redundant features. Figure 5 depicts the importance distribution of spectral and image features. The greater the weight, the more important the corresponding features. According to sequential backward elimination, all features with weights greater than 0.2 were selected to detect wheat samples with mild, moderate, and severe infection; the result was five spectral features, three texture features, and two color features (Table 3). The weights of the selected spectral features were much higher than remaining spectral features (Figure 5A). One selected spectral feature was located in the visible region, three were located in the red edge region, and one was located in the near-infrared region. For the image features, three texture features and two color features were selected to illustrate the distributions of disease and the degree of infection in wheat; the maximum weight of selected image features reached 0.39. Table 4 demonstrates the separation ability of these selected features to detect mild, moderate, and severe samples by ANOVA. In general, the selected features show different mean and standard deviation values among multi-class samples. There were significant differences among the mild, moderate, and severe samples of all features, and the significance level reached 0.95. Therefore, the selected features have strong separation ability to detect infected samples in this study.

**Table 3:** The features selected by importance ranking.

Type	Variable number	Selected Features
Spectral features	5	band1(518 nm), band2(666 nm), band3(706 nm), band4(742 nm) and band5(846 nm)
Texture features	3	mean1, mean3 and hom3
Color features	2	MGRVI and NGRDI

**Table 4:** Statistical characteristics of feature values of the mild, moderate, and severe disease samples.

Feature	Sample category	Mean of feature	Std. deviation	*P*-Value (ANOVA)
band1	Mild	0.059	0.013	0.002
	Moderate	0.063	0.014	
	Severe	0.076	0.011	
band2	Mild	0.054	0.017	0.035
	Moderate	0.057	0.019	
	Severe	0.071	0.016	
band3	Mild	0.155	0.041	0.001
	Moderate	0.168	0.039	
	Severe	0.209	0.030	
band4	Mild	0.312	0.070	0.000
	Moderate	0.345	0.065	
	Severe	0.418	0.054	
band5	Mild	0.374	0.084	0.000
	Moderate	0.411	0.079	
	Severe	0.496	0.062	
mea1	Mild	21.88	3.383	0.038
	Moderate	21.32	4.067	
	Severe	18.56	2.238	
mea3	Mild	37.03	11.207	0.003
	Moderate	32.66	10.748	
	Severe	24.12	6.161	
hom3	Mild	0.78	0.112	0.019
	Moderate	0.80	0.083	
	Severe	0.76	0.100	
MGRVI	Mild	−0.32	0.037	0.031
	Moderate	−0.35	0.059	
	Severe	−0.40	0.046	
NGRDI	Mild	−0.16	0.024	0.030
	Moderate	−0.18	0.032	
	Severe	−0.21	0.027	

### Model construction

The purpose of our study is to effectively identify field FHB based on the fusion of spectral and image features of UAV images to be able to control the development of field diseases in a timely manner. Therefore, the classification models were developed by combining different feature fusion with SVM, RF, and BPNN for the analysis of wheat FHB detection. The calibration accuracy and prediction accuracy of the models are shown in Table 5. The precisions of models constructed based on different feature variables are significantly different. The integration of spectral, texture, and color features seems to achieve the best accuracy. When spectral features were used as model inputs, the RF model performed best with a prediction accuracy of 70%, followed by BPNN and SVM with prediction accuracies of 65 and 60%, respectively. When considering the integration of spectral and texture features, the accuracy of the three classification models was improved by 10%, and the RF model achieved the highest accuracy at 80%. When spectral, texture, and color features were integrated as input variables, the prediction accuracy of the RF model was further improved to 85%. The prediction accuracy of the SVM model remained unchanged, but the prediction accuracy of the BPNN model was also improved by 5%. The calibration accuracy of the model also shows the same trend as the prediction accuracy. Among all models, the calibration accuracy of the RF model reached 100%. With the addition of image features, the calibration accuracy of the model continued to improve. The above results indicate that the fusion of spectral and image features can improve the performance of the model through texture and color features in terms of identifying wheat FHB. The five-fold cross-validation method was used to further verify the model to prove its universality. The validation results are shown in Table 5. The results show that the highest validation accuracy was 83%, which is reflected in the integration of spectral feature, texture feature, color feature, and RF algorithm. The above results show that the spectral and image feature fusion combined with the RF algorithm can benefit the rapid detection and accurate analysis of a wheat field with mild, moderate, and severe infection.

**Table 5:** Model classification accuracy based on different features and algorithms.

Feature	Classification algorithm	Calibration accuracy (%)	Prediction accuracy (%)	Validation accuracy (%)
Spectral	RF	100	70	70
	SVM	63	60	59
	BPNN	78	65	72
Spectral + texture	RF	100	80	79
	SVM	70	70	60
	BPNN	76	75	76
Spectral + texture + color	**RF**	**100**	**85**	**83**
	SVM	74	70	63
	BPNN	84	80	83

Bold values indicate the optimal algorithm and highest accuracy.

**Figure 6:**
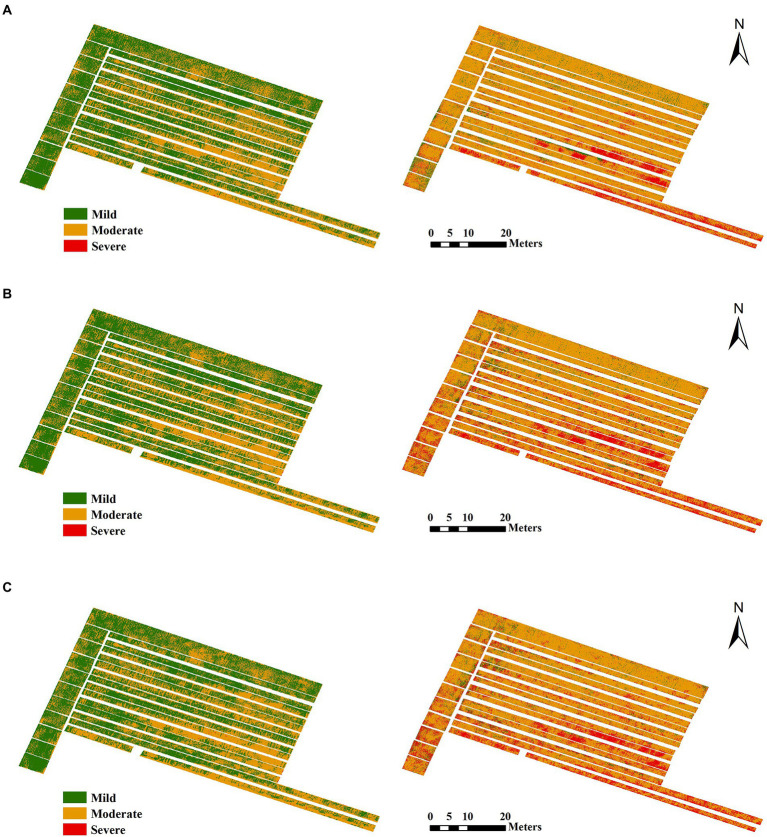
Damage maps for May 3 (left) and May 8 (right) based on different feature combinations and the RF algorithm. **(A)** Spectral features. **(B)** Spectral and texture features. **(C)** Spectral, texture, and color features.

To understand the spatial distribution of FHB-infected wheat in the study area, models based on different feature integrations and the optimal RF algorithm were adapted to map the damage of wheat FHB on May 3 and 8, 2019. The results are shown in Figure 6. From the mapping results, the wheat infection degree increased over time. Although the infection had spread over the entire farmland on May 3, the wheat in the field showed mild and moderate infection, and severe infection was almost zero (sporadic distribution). However, on May 8, almost all wheat in the study area showed moderate or severe infection, indicating that a large outbreak rapidly occurred. Table 6 summarizes the proportions of wheat area with mild infection, moderate infection, and severe infection corresponding to each figure in Figure 6. Moderate infection impacted more than 75% of the wheat on May 8, and the severe infection impacted more than 10%. The addition of image features improved the model in terms of detecting severely infected wheat. The proportion of severely infected wheat on May 8 in Figure 6B (18.12%) and Figure 6C (18.85%) is higher than that in Figure 6A (11.73%); these results are mainly reflected in the presence of some severe infection along the edge of the plot. This severe infection phenomenon is consistent with our field survey results.

**Table 6:** The percentages of mildly, moderately, and severely infected wheat corresponding to the damage maps.

Feature	Data	Mild (%)	Moderate (%)	Severe (%)	Sum (%)
Spectral	May 3	57.16	42.76	0.08	100
	May 8	5.72	82.55	11.73	100
Spectral + texture	May 3	55.45	44.45	0.10	100
	May 8	5.67	76.21	18.12	100
Spectral + texture + color	May 3	53.82	46.11	0.08	100
	May 8	5.26	75.88	18.85	100

## Discussion

In the present work, the detailed information contained in UAV hyperspectral images were fully exploited to help identify wheat FHB in the field. FHB can change the pigment, water content, and cell structure of wheat, as well as the structure, shape, and color of the wheat canopy. Therefore, we fused the spectral features that represent internal physiological changes with the image features that represent the spatial information of wheat to effectively detect wheat FHB.

Before analyzing the UAV images, we first evaluated the quality of the UAV hyperspectral images, which is a critical step to ensure that the UAV images accurate identify FHB. We evaluated the quality of the UAV images by comparing and analyzing the data obtained from an ASD spectrometer. The spectral curves of the wheat sample points extracted from the UAV images share a common trend with those of the ASD spectral: a peak and a trough in the VIS–NIR region. However, the values of the ASD spectra were lower than the spectral values obtained from the UAV images, which is likely due to the influence of the bidirectional reflectance distribution function (BRDF) caused by the difference in the geometrical positions of the sun-target-sensors of the two data sets. Some studies have proven that BRDF has a significant impact on UAV hyperspectral data (Burkart et al., 2015). Above 850 nm, the UAV spectral curve shows a downward trend compared with the ASD spectral curve, while the ASD spectral curve has little variation, which is consistent with other scholars’ observations (Gao et al., 2016; Chen et al., 2018). The sensor may have too much noise at the detection boundary. Furthermore, it need cloud-free conditions on the measurement day, so there is a long time interval between the UAV flight and ASD information collection, as well as changes in light conditions. According to Figure 4, the spectral reflectance of sample points obtained by different sensors is significantly correlated within 450–850 nm (*R*^2^ of 0.99). ASD hyperspectral data are an extensive remote sensing identification method for crop pests and diseases (Cao et al., 2013; Shi et al., 2018; Guo et al., 2020). This spectrum has been used to accurately identify field wheat FHB (Huang et al., 2019a,b; Ma et al., 2020). The high correlation between UAV and ASD spectra further proves the reliability of UAV images.

Next, we extracted the band features, texture features, and color features contained in the hyperspectral images. Table 3 shows the details of the extracted features. The band features we extracted are mainly located in the green edge, red edge, and near-infrared region. The green edge is mainly related to the content of wheat pigments (including carotenoids and chlorophyll) (Al Masri et al., 2017; Zhang Z. P. et al., 2020b), and the position of the red edge is sensitive to the movement of the red edge caused by the change of chlorophyll concentrations (Zhang Z. P. et al., 2020a). Near-infrared wavelengths are primarily related to wheat moisture content, as FHB-infected wheat is accompanied by a temporary increase in transpiration and tissue desiccation (Bauriegel et al., 2011a). The VIS–NIR bands in hyperspectral images are proposed to overcome visual symptom disassociations with DON contamination. Because the DON concentration of wheat is at a low level in the early stages and the typical symptoms of *Fusarium* damage cannot be detected visually, the spectral features are more conducive to observing the early symptoms of wheat infection (Femenias et al., 2020; Zhang et al., 2021). The GLCM-based texture feature extraction method was based on Fu et al. (2022). The GLCM method describes the texture by studying the spatial correlation characteristics of the gray levels (Haralick and Shanmugam, 1973). In fact, texture information can help distinguish the spatial information independent of tone to identify objects or regions of interest in an image, but it is not recommended to use it by itself due to the poor performance of texture parameters (Sarker and Nichol, 2011). Previously, auxiliary texture information was effectively combined with spectral information to significantly improve the accuracy of wheat GPC estimation (Fu et al., 2022). Therefore, in this study, we attempted to fuse texture and spectral features to improve the detection accuracy of field FHB. The results demonstrate that texture features can serve as complementary information to increase the dimensionality of UAV hyperspectral image data (Table 5). In addition, we calculated some color features by band combinations to indicate different aspects of wheat infection. While texture features may add additional information to FHB estimation, crop infection is more directly related to color information rather than the spatial arrangement of colors (Li et al., 2019). What’s more, since color images highlight specific vegetation greenness and are considered to be less sensitive to changes in light conditions, color features extracted from RGB images have the potential to provide crop growth and nutritional status, immediately providing researchers and farmers with a realistic and intuitive visualization of crop growth status (Du and Noguchi, 2017; Ge et al., 2021). At present, some scholars use color features to estimate the nitrogen density of winter wheat leaves (Rorie et al., 2011), estimate the leaf area index of rice (Li et al., 2019), monitor the growth status of wheat (Du and Noguchi, 2017), and accurately detect wheat FHB at the spikes scale (Huang et al., 2020). However, the effect of FHB detection of field wheat based on color features has not been explored yet. Therefore, in this study, we further supplemented color features in the input models based on spectral and texture features to identify wheat FHB. Actually, color features in UAV digital images are usually based on RGB cameras because UAV systems with RGB cameras are inexpensive, compact, and convenient. In the future, a UAV system suitable for FHB monitoring in the field should be considered. The RGB bands in this study are a basis for future RGB cameras, avoiding the complexity of hyperspectral data processing.

Table 5 shows the model classification results of field wheat with different degrees of infection according to different input variables. The addition of texture and color features can further improve the accuracy of the model compared to methods that use spectral features to detect wheat FHB. As seen in Table 6, the improvement of accuracy is mainly manifested in the difference in the model’s detection of mild, moderate, and severe disease samples. In the early stage of wheat FHB infection (May 3), 57.16% and 42.76% of the field wheat with mild and moderate infection, respectively, could be identified by the model using spectral features. With the addition of texture features and color features, the proportion of mildly infected wheat in the field identified by the model gradually decreased, and the proportion of moderately infected wheat increased. In the late stage of wheat FHB infection (May 8), the model indicated that the proportions of mildly and moderately infected wheat gradually decreased with the addition of image features, and the severely infected area gradually increased. That is to say, before the image features are added, the model always misses the wheat with more severe disease. In fact, as the wheat infection spread, the dry and white areas of the wheat ears became larger until the wheat died (Huang et al., 2020). The addition of image features can enable the model to capture this process. When the information contained in the model increases, the detection of samples with severe disease improves, which is consistent with the results in our study.

The research shows that the fusion of spectral and image features can distinguish the disease incidence of wheat in the field; this method can help future precision agriculture and large area wheat FHB monitoring. However, current research still exists limitations. In addition to considering spectral features, texture features, and color features, some vegetation features, such as Structure Insensitive Pigment Index (SIPI), Anthocyanin Reflectance Index (ARI), Normalized Difference Vegetation Index (NDVI), and Plant Senescence Reflection Index (PSRI), are often used to reflect plant disease stress status (Xiao et al., 2021). Hence, the effectiveness of the vegetation features in wheat FHB detection based on UAV hyperspectral images is worth considering. Additionally, only three machine learning algorithms (RF, BPNN, and SVM) were used in this study. The generalization ability of the models in the temporal and spatial dimensions must be verified. Further consideration can be given to combining data augmentation and deep learning methods to develop more stable and independent models, as well as reduce the uncertainty of model applicability in other regions. Scale has become a popular topic in remote sensing research, and the information contained in a single pixel under different resolutions will change significantly. Appropriate spatial resolution images for agricultural monitoring are needed (Na et al., 2016). Our study only used two images with a spatial resolution of 4 cm to detect FHB, which is relatively simple. Various spatial resolution images are worth considering in the future. Finally, the occurrence of wheat FHB is related to the time of infection (Alisaac et al., 2020) and meteorological factors, such as temperature and humidity. In the future, we will aim to consider wheat infection time and meteorological factors to explore early FHB detection methods and effectively prevent and control FHB occurrence and outbreak. The influence of wheat varieties and the development of various pests and diseases on the spread of wheat FHB cannot be ignored. More researches are needed to investigate the influence of varieties and multiple infections on the model performance in the future.

## Conclusion

In this study, the quantitative detection of wheat with mild, moderate, and severe FHB infection in the field was achieved by fusing spectral and image features extracted from the UAV hyperspectral images. After obtaining the hyperspectral images, we first evaluated the quality of the images and identified the data in the 450–850 nm band for subsequent analysis by comparing waveform similarity and correlation with ASD hyperspectral data. Then, we extracted the spectral features that reflect the physiological and biochemical changes within the host, as well as the texture and color features that characterize the spatial changes of wheat. The RF algorithm was used to further eliminate redundant features and improve the operating efficiency of the model. Finally, FHB quantitative detection models, based on different combinations of spectral features, texture features, and color features were formulated by combining BPNN, SVM, and RF algorithms. We evaluated the classification results of the different models, and the FHB-related wheat damage was mapped using the best algorithm. The results show that the spectral features can potentially determine the damage level of FHB, but the performance of the models is not satisfactory. The fusion of spectral features and texture features can improve the model detection level, but the maximum prediction accuracy of the models was only 80%. The model based on the fusion of spectral, texture, and color features was best, and the prediction accuracy of the RF algorithm reached 85%. The damage map illustrates that wheat FHB developed very rapidly over a short time, causing destruction of the crop. This study builds upon previous models in terms of feature types, monitoring methods, and monitoring areas and provides a new methodology for FHB detection in the field by deeply mining features in UAV images and combining multiple spectral advantages.

## Data availability statement

The raw data supporting the conclusions of this article will be made available by the authors, without undue reservation.

## Author contributions

HZ: data curation, methodology, and writing-original draft. HM and LL: investigation and data acquisition. JZ and SW: formal analysis, methodology, and supervision. LH: conceptualization and funding acquisition. YD and WH: writing-review and editing. All authors contributed to the article and approved the submitted version.

## Funding

The work presented here was supported by National Key R&D Program of China (2021YFB3901303), National Natural Science Foundation of China (42071423), Program of Bureau of International Cooperation, Chinese Academy of Sciences (183611KYSB20200080), Alliance of International Science Organizations (Grant No. ANSO-CR-KP-2021-06), Natural Science Research Project of Anhui Provincial Education Department (KJ2019A0030).

## Conflict of interest

The authors declare that the research was conducted in the absence of any commercial or financial relationships that could be construed as a potential conflict of interest.

## Publisher’s note

All claims expressed in this article are solely those of the authors and do not necessarily represent those of their affiliated organizations, or those of the publisher, the editors and the reviewers. Any product that may be evaluated in this article, or claim that may be made by its manufacturer, is not guaranteed or endorsed by the publisher.
